# Supplementary material for: Prognostic Value of Coronary Calcium Score in Asymptomatic Individuals: A Systematic Review
Source: J Clin Med. 2022 Oct 1;11(19):5842. doi: 10.3390/jcm11195842 (PMC9573072; doi:10.3390/jcm11195842)
Supplement: Supplementary file 1 [file jcm-11-05842-s001.zip › jcm-1935520-supplementary.pdf]

Prognostic value of Coronary Calcium Score in asymptomatic individuals: a systematic review

Liberatore Tramontano[1]; Bruna Punzo[1]; Alberto Clemente[2]; Sara Seitun[3]; Luca Saba[4]; Eduardo Bossone[5]; Erica Maffei[2]; Carlo Cavaliere[1]; Filippo Cademartiri[2*]

[1] IRCSS SYNLAB SDN; Naples, Italy
[2] Department of Radiology, Fondazione Monasterio/CNR, Pisa, Italy
[3] Department of Radiology, IRCCS Ospedale San Martino, Genoa, Italy
[4] Department of Radiology, University of Cagliari, Cagliari, Italy
[5] Department of Cardiology, Azienda Ospedaliera Antonio Cardarelli, Naples, Italy
* Correspondence: fcademartiri@ftgm.it; Prof. Dr. Filippo Cademartiri, MD, PhD, FESC; Chairman of Radiology - Fondazione Toscana "Gabriele Monasterio"; Ospedale "San Cataldo" di Pisa - Area della Ricerca CNR (Consiglio Nazionale delle Ricerche); via Giuseppe Moruzzi, 1 - 56124 - Pisa (PI) – Italy

Supplementary Materials

Table S1. Key terms used in literature search.

- calcium score [Title/Abstract]) AND (computed tomography[Title/Abstract] AND (prognostic [Title/Abstract])) AND (asymptomatic [Title/Abstract])
- calcium score [Title/Abstract]) AND (prognostic [Title/Abstract]))
- calcium score AND computed tomography AND prognostic AND asymptomatic
- TITLE-ABS (calcium score) AND TITLE-ABS (prognostic) AND TITLE-ABS (asymptomatic) AND TITLE-ABS (computed AND tomography) OR TITLE-ABS (ct)

Table S2. Prisma checklist.

Section/topic	#	Checklist item	Reported on page #
**TITLE**			
Title	1	Identify the report as a systematic review, meta-analysis, or both.	1
**ABSTRACT**			
Structured summary	2	Provide a structured summary including, as applicable: background; objectives; data sources; study eligibility criteria, participants, and interventions; study appraisal and synthesis methods; results; limitations; conclusions and implications of key findings; systematic review registration number.	2
**INTRODUCTION**			
Rationale	3	Describe the rationale for the review in the context of what is already known.	3-4
Objectives	4	Provide an explicit statement of questions being addressed with reference to participants, interventions, comparisons, outcomes, and study design (PICOS).	3-4
**METHODS**			
Protocol and registration	5	Indicate if a review protocol exists, if and where it can be accessed (e.g., Web address), and, if available, provide registration information including registration number.	NA
Eligibility criteria	6	Specify study characteristics (e.g., PICOS, length of follow-up) and report characteristics (e.g., years considered, language, publication status) used as criteria for eligibility, giving rationale.	4-5
Information sources	7	Describe all information sources (e.g., databases with dates of coverage, contact with study authors to identify additional studies) in the search and date last searched.	4-5
Search	8	Present full electronic search strategy for at least one database, including any limits used, such that it could be repeated.	4-5
Study selection	9	State the process for selecting studies (i.e., screening, eligibility, included in systematic review, and, if applicable, included in the meta-analysis).	4-5
Data collection process	10	Describe method of data extraction from reports (e.g., piloted forms, independently, in duplicate) and any processes for obtaining and confirming data from investigators.	4-5
Data items	11	List and define all variables for which data were sought (e.g., PICOS, funding sources) and any assumptions and simplifications made.	4-5
Risk of bias in individual studies	12	Describe methods used for assessing risk of bias of individual studies (including specification of whether this was done at the study or outcome level), and how this information is to be used in any data synthesis.	4-5
Summary measures	13	State the principal summary measures (e.g., risk ratio, difference in means).	NA
Synthesis of results	14	Describe the methods of handling data and combining results of studies, if done, including measures of consistency (e.g., I^2) for each meta-analysis.	NA

Section/topic	#	Checklist item	Reported on page #
Risk of bias across studies	15	Specify any assessment of risk of bias that may affect the cumulative evidence (e.g., publication bias, selective reporting within studies).	NA
Additional analyses	16	Describe methods of additional analyses (e.g., sensitivity or subgroup analyses, meta-regression), if done, indicating which were pre-specified.	NA
**RESULTS**			
Study selection	17	Give numbers of studies screened, assessed for eligibility, and included in the review, with reasons for exclusions at each stage, ideally with a flow diagram.	6
Study characteristics	18	For each study, present characteristics for which data were extracted (e.g., study size, PICOS, follow-up period) and provide the citations.	7-15
Risk of bias within studies	19	Present data on risk of bias of each study and, if available, any outcome level assessment (see item 12).	16
Results of individual studies	20	For all outcomes considered (benefits or harms), present, for each study: (a) simple summary data for each intervention group (b) effect estimates and confidence intervals, ideally with a forest plot.	NA
Synthesis of results	21	Present results of each meta-analysis done, including confidence intervals and measures of consistency.	NA
Risk of bias across studies	22	Present results of any assessment of risk of bias across studies (see Item 15).	NA
Additional analysis	23	Give results of additional analyses, if done (e.g., sensitivity or subgroup analyses, meta-regression [see Item 16]).	NA
**DISCUSSION**			
Summary of evidence	24	Summarize the main findings including the strength of evidence for each main outcome; consider their relevance to key groups (e.g., healthcare providers, users, and policy makers).	16-18
Limitations	25	Discuss limitations at study and outcome level (e.g., risk of bias), and at review-level (e.g., incomplete retrieval of identified research, reporting bias).	19
Conclusions	26	Provide a general interpretation of the results in the context of other evidence, and implications for future research.	19
**FUNDING**			
Funding	27	Describe sources of funding for the systematic review and other support (e.g., supply of data); role of funders for the systematic review.	NA

From: Moher D, Liberati A, Tetzlaff J, Altman DG, The PRISMA Group (2009). Preferred Reporting Items for Systematic Reviews and Meta-Analyses: The PRISMA Statement. PLoS Med 6(7): 1000097. doi:10.1371/journal.pmed1000097[28].

Table S3. Quality assessment using QUIPS tool.

Study	Year	Study participation	Study Attrition	Prognostic Factor Measurement	Outcome Measurement	Study Confounding	Statistical analysis and reporting
Dzaye O. et al	2020	Moderate	Low	Low	Low	Low	Low
Blaha et al	2020	Moderate	Low	Low	Low	Low	Low
Dudum R. et al	2019	Moderate	Low	Low	Low	Low	Low
Huang ZL. et al	2019	Moderate	Low	Low	Low	Low	Low
Lahti SJ. et al	2019	Moderate	Low	Low	Low	Low	Low
Serra CM. et al	2019	Low	Low	Low	Low	Low	Low
Shaikh et al	2019	Low	Low	Low	Low	Low	Low
Moon SJ et al	2019	Low	Low	Low	Low	Moderate	Low
Cho I. et al	2018	Low	Low	Low	Low	Low	Low
Orimoloye et al	2018	Low	Low	Low	Low	Low	Low
Han D. et al	2018	Low	Low	Low	Low	Moderate	Low
Malik S. et al	2017	Low	Low	Low	Low	Low	Low
Palmieri V. et al	2017	Moderate	Low	Low	Low	Low	Moderate
Cho et al	2017	Low	Low	Low	Low	Low	Low
Carr et al	2017	Low	Low	Low	Low	Low	Low
Takamura K. et al	2017	Moderate	Low	Low	Low	Low	Low
Choi SY. et al	2016	Moderate	Low	Low	Low	Moderate	Low
Radford NB et al	2016	Low	Low	Low	Low	Low	Low
Valenti et al	2016	Low	Low	Low	Low	Low	Low
Lee et al	2016	Moderate	Low	Low	Low	Low	Low
Kelkar et al	2016	Low	Low	Low	Low	Low	Low
Knapper et al	2016	Low	Low	Low	Low	Moderate	Low
Dedic et al	2016	Moderate	Moderate	Low	Low	Moderate	Low
Halon D.A. et al	2016	Low	Low	Low	Low	Moderate	Low
van den Hoogen IJ. et al	2016	Low	Low	Low	Low	Low	Low
Chang SM. et al	2015	Low	Low	Low	Low	Moderate	Low
Dikic M. et al	2015	Low	Low	Low	Low	Low	Low
Han D. et al	2015	Moderate	Low	Low	Low	Low	Low
Havel M. et al	2015	Low	Low	Low	Low	Moderate	Low
Valenti V. et al	2015	Low	Low	Low	Low	Low	Low
Shaw et al	2015	Moderate	Low	Low	Low	Low	Low
von Sholten et al	2015	Low	Low	Low	Low	Moderate	Low
Cho I. et al	2015	Low	Low	Low	Low	Moderate	Low
Hur J et al	2015	Low	Low	Low	Low	Moderate	Low
Faustino A. et al	2014	Low	Low	Low	Low	Moderate	Low
Patel J. et al	2014	Low	Low	Low	Low	Low	Low
Plank F. et al	2014	Low	Low	Low	Low	Moderate	Low
K. Min et al	2014	Low	Low	Low	Low	Low	Low

Park et al	2013	Moderate	Low	Low	Low	Low	Low
Graham G. et al	2012	Low	Low	Low	Low	Low	Low
McEvoy et al	2012	Low	Low	Low	Low	Low	Low
Nasir K. et al	2012	Low	Low	Low	Low	Low	Low
Rana et al	2012	Low	Low	Low	Low	Low	Low
Shemesh J. et al	2011	Moderate	Low	Low	Low	Low	Low
Yoo DH. et al	2011	Moderate	Moderate	Low	Moderate	Moderate	Low
